# Synergistic Effects of Sugarcane Bagasse Biochar and *Eisenia fetida* Enhance Biogenic Stabilization of Sewage Sludge and Sugar Industry Waste Through Vermicomposting

**DOI:** 10.3390/biology15080622

**Published:** 2026-04-16

**Authors:** Muhammad Bilal Khan, Xiaoqiang Cui, Qi Tao, Yasir Hamid, Bilal Hussain, Zeeshan Zafar, Zhenli He, Xiaoe Yang

**Affiliations:** 1 Key Laboratory of Environmental Remediation and Ecological Health, Ministry of Education, College of Environmental and Resource Sciences, Zhejiang University, Hangzhou 310058, China; cuixiaoqiang@tju.edu.cn (X.C.);; 2 Soil and Water Science Department, Indian River Research and Education Center, Institute of Food and Agricultural Sciences, University of Florida, Fort Pierce, FL 34945, USA; 3 School of Environmental Science and Engineering, Tianjin University, Tianjin 300072, China; 4 College of Resources, Sichuan Agricultural University, Chengdu 611130, China; 5 State Key Laboratory of Water Resources Engineering and Management, Wuhan University, Wuhan 430072, China

**Keywords:** biochar amendment, vermicomposting, biowaste valorization, heavy metal stabilization, organic fertilizer, *Eisenia fetida*

## Abstract

Integrated waste management through vermicomposting combined with biochar amendments represents an innovative approach for sustainable resource recovery. This study evaluated the effects of sugarcane bagasse biochar (SBB) at 0%, 5%, and 10% application rates on *Eisenia fetida* performance and vermicompost quality during preincubation-vermicomposting of sewage sludge and press-mud mixtures. The 10% SBB treatment significantly (*p* < 0.05) enhanced earthworm biomass (72.3% increase) and cocoon production (24.8 ± 1.8 per earthworm vs. 12.3 ± 1.2 in control). Lignocellulosic degradation improved substantially, achieving 22.6%, 10.7%, and 38.8% degradation for cellulose, hemicellulose, and lignin, respectively. Macronutrient concentrations increased significantly: TN by 38.4%, TP by 15%, and TK by 21.4% compared to initial mixtures. Moreover, total heavy metal concentrations decreased significantly during vermicomposting, with reductions of 8.1–8.7% for Pb, 5.3–7.6% for Cd, and 3.0–4.8% for Cr, with reduced bioavailability factors indicating enhanced metal stabilization. The final vermicompost exhibited optimal maturity indices, including a C:N ratio of 15.4 ± 0.2 and improved electrical conductivity. Results demonstrate that 10% sugarcane bagasse biochar amendment facilitates efficient concurrent management of sewage sludge and sugarcane industrial wastes while producing high-quality organic fertilizer with enhanced nutrient content, reduced heavy metal bioavailability, and accelerated stabilization for sustainable agricultural/horticultural applications.

## Simple Summary

This study investigated a simple and eco-friendly way to manage different types of biowaste by using earthworms and biochar (a charcoal-like material made from sugarcane bagasse). Sewage sludge and sugarcane press mud were mixed and treated with different amounts of sugarcane bagasse biochar. When 10% biochar was added during preincubation, earthworms grew better and produced more cocoons, meaning the vermicomposting process worked more efficiently. The waste broke down faster, important plant nutrients increased, and harmful heavy metals were reduced and became less available. In the end, the process produced a safe, stable, and nutrient-rich organic fertilizer that can be used for horticultural purposes, showing a practical way to turn various biowastes into a useful product.

## 1. Introduction

China is the most populous country globally and the largest consumer of food. China is feeding 20% of the world’s human population, which consumes around 5 million tons of food yearly. China is also the leading agricultural producing country, having 36% of the world’s total agricultural land covered [1]. Over the past two decades, a vast quantity of solid waste has been produced worldwide, which might be due to several factors such as the escalation in global population, urbanization, and changes in consumption behavior. Biowastes consist of several types of wastes, i.e., yard waste, food waste, process residues, and agricultural wastes, consisting of a large proportion (46%) of the worldwide production of solid waste recycling [2]. According to Khan et al. (2019), average sewage sludge production in the world is 20–40 kg per population equivalent per year [3]. In recent years, China has also seen an increase in sewage sludge production (60 million tons), which may be related to the country’s fast urbanization, population growth, and increase in the number of wastewater treatment plants [3]. Sewage sludge comprises many detrimental organic and inorganic toxins, including toxic metals and different pathogens. Therefore, direct disposal of sewage sludge into the environment without any treatment discharges contaminants into the environment that could pass through the food chain and eventually pose many detrimental threats to human health. Consequently, the appropriate recycling and valorization of sewage sludge is very important for the safe disposal of sewage sludge into the environment.

A large quantity of agricultural waste is being produced annually throughout the world due to massive agricultural activities to fulfill the demands of food for an increasing population. Agricultural waste is a serious issue, especially in Asian countries such as China, Pakistan, and India, where there is growing concern about how to efficiently manage and dispose of this organic refuse. The main agricultural crops of China are rice, wheat, corn, and sugarcane, which produce a large quantity of lignocellulosic agricultural wastes. The current global production of sugarcane (*Saccharum officinarum*) is approximately 1.1 billion tons annually, and China is producing around 123 million tons of sugarcane yearly [4]. Brazil and India are the world’s top producers of sugarcane, accounting for 33% and 22.4% of total production, respectively. Other major producers of sugarcane are China, Thailand, Pakistan, Colombia, Mexico, and Australia, which together account for 22.7% of the world’s total production. In China, approximately 80% of total sugar production is produced from the sugarcane crop. The area under sugarcane cultivation expanded dramatically, increasing 13.8-fold from 0.12 million hectares in 1949 to 1.7 million hectares in 2013. Correspondingly, sugar production rose by 44.5 times, from 0.28 million tons in 1949 to 12.5 million tons in 2013 [5]. Sugarcane is an intensive crop, which accumulates enormous amounts of plant nutrients from the soil during growth, mainly nitrogen, phosphorus, and potassium. The average amount of plant nutrients depleted from soil for 100 MT of sugarcane production is N, P, K, Ca, Mg, S, Fe, Mn, Zn, and Cu, i.e., 148, 123, 238, 42, 39, 38, 7.5, 4.1, 0.5, and 0.1 kg, respectively. During the production of sugarcane crops, large quantities of plant nutrients are being depleted from the soil ecosystem and dumped in the surrounding area of sugarcane industries as industrial waste. This sugarcane industrial waste consists mainly of bagasse, press mud, and trash, having an adequate quantity of macro and micronutrients, which are vital for the growth of plants. These plant nutrients present in sugarcane industrial wastes can be successfully salvaged for the enhancement of soil fertility and crop production. One of the major sugarcane industrial wastes is sugarcane bagasse (SB), which is a highly lignocellulosic and fibrous material acquired after removing the juice from sugarcane in sugar and alcohol industries [6]. The worldwide production of SB exceeds 540 million tons annually [7], while the annual production of SB in China is approximately 5.2 million tons [5]. Similar to most agricultural wastes, SB is a carbonaceous material, produced in large quantities and appropriate for biofuel and biochar production. Being a complex lignocellulosic material, sugarcane bagasse is mostly composed of 50% cellulose, 25% hemicellulose, and 25% lignin. It also contains pentosans, β-cellulose, and ash [8]. Sugarcane bagasse has substantial potential for second-generation biofuel production [9], recycled in distillery plants for energy production [8], destined for bioplastic transformation, and used for generating electricity [5]. Furthermore, SB can also be used to produce chemical substances such as paper paste, furfural, hydroxymethyl furfural, or ethanol [10]. It is wise to employ this enormous amount of industrial waste in the development of environmentally friendly technologies like composting and vermicomposting, as sugarcane is produced in nearly 80 countries worldwide. According to previous studies, lignocellulosic sugarcane waste can be effectively converted to high-value organic fertilizers [11]. Furthermore, sewage sludge was mixed in combination with various supplementary materials, i.e., food waste, anaerobically digested biogas plant slurry, garden waste, mature vermicompost, vinasse bio-waste, sugarcane trash, rabbit manure, sawdust, straw, hay, pine bark, and paper mulch [3]. Some other supplementary materials used during vermicomposting and studied by different researchers include bulking agents such as tea factory coal ash, fly ash, phosphate rock, and biochar [12]. These bulking agents can be combined with organic waste either at the commencement or conclusion of the composting process, after which vermicomposting may commence. Sugarcane bagasse and press mud are easily available in large quantities, especially in countries like Brazil and China, because these are big producers of the sugarcane crop. Additionally, the use of sugarcane bagasse for ecofriendly purpose would reduce industrial waste and negative impact on the environment [13]. Sugarcane bagasse can be used for making sugarcane bagasse biochar (SBB) through the pyrolysis of the anaerobic digestion process [14]. Previous studies showed that SBB has been used to ameliorate the quality of soil and water and elevate the growth of plants [15]. These advantages of SBB are due to the high surface area and occurrence of different chemical functional groups [4]. Recent work has emphasized the importance of carbon-based amendments in enhancing organic waste stabilization and improving nutrient and contaminant dynamics during biological treatment processes [16]. This aligns with the growing interest in using biochar, such as sugarcane bagasse biochar, to improve substrate structure, support microbial activity, and reduce pollutant bioavailability in vermicomposting systems. Until now, there is no information available in published literature on the effect of SBB on earthworm growth, reproduction, survival, and vermicompost quality during vermicomposting of organic wastes. This experiment aimed to investigate the effects of SBB on *Eisenia fetida* growth, reproduction, and survival, as well as the quality of vermicompost generated during vermi-stabilization of sewage sludge and waste from the sugarcane industry.

## 2. Materials and Methods

### 2.1. Collection of Waste Materials

Fresh sewage sludge (SS) was collected from the nearby municipal wastewater treatment plant of Linan City, China. The collected SS was air-dried in open plastic boxes for seven days with manual turning. Different non-decomposable materials, such as plastic parts, metal parts, glass pieces, rubber bands, and chewing gum, etc., were separated. Sugarcane bagasse (SB) and press mud (PM) were collected from Guangxi Shanglin Sugar Industry, Nanning Shanglin County, Guangxi Province, China. Sugarcane bagasse was sundried for one week with manual turning and was chopped into small bits (>1 cm). The Physiochemical properties of SS, PM, and SSB are presented in Table 1. For the vermicomposting experiment, earthworms (*Eisenia fetida*) were obtained from a commercial seller, and a nursery of earthworms (*Eisenia fetida*) was raised by using cow manure waste in the greenhouse of Zhejiang University, China.

### 2.2. Preparation of Sugarcane Bagasse Biochar (SBB)

Sugarcane bagasse for the preparation of biochar was washed with distilled water, air dried by spreading on a plastic sheet, and finally oven dried at 80 °C for two days with the intention of decreasing the moisture. The oven-dried SB was then subjected to grinding by using a stainless grinding machine MM 400 (RETSCH GmbH, Haan, Germany), and then the powder material was sieved to <2.0 mm. The powder biomass was then put into a ceramic pot, and pyrolysis was done by using a muffle furnace under a nitrogen atmosphere. The detailed process of pyrolysis is already given in our previously published research work [12]. At the end of the pyrolysis process, the received biochar was referred to as sugarcane bagasse biochar (SBB). Before being used for the experiment, SBB was finally crushed to pass through a 0.5 mm sieve.

### 2.3. Morphological Assessment

The surface and structural characteristics of SBB were compared using scanning electron microscopy (SEM) imaging investigations utilizing a FEI Quanta FEG 650 scanning electron microscope (FEI Company, Hillsboro, OR, USA). Energy-dispersive X-ray spectroscopy (EDS) system, Genesis XM (EDAX Inc., Mahwah, NJ, USA) was used at the same location to perform elemental analysis from the surfaces of SBB, initial mixture, and final vermicompost samples using SEM. Additionally, 50 images at a resolution of 2 cm^−1^ were used in conjunction with Fourier transform infrared (FTIR) analysis to classify the surface functional chemical groups using a Nicolet 6700 FT-IR spectrometer (Thermo Fisher Scientific, Waltham, MA, USA) in the range of 400–4000 cm^−1^ [3].

### 2.4. Experimental Setup

#### 2.4.1. Preincubation Experiment

A 16-day preincubation experiment was carried out in round, 60 L composting bioreactors in a greenhouse. The exact specifications for the preincubation reactor are already provided by our previously published work [3]. A homogenized mixture of SS and PM in an 80:20 ratio was created at the beginning of the preincubation experiment, and 0%, 5%, and 10% of SBB were added to each preincubation bioreactor. SS + PM + NB (33.6 + 8.4 + 0), SS + PM + 5% SBB (33.6 + 8.4 + 2.1), and SS + PM + 10% SBB (33.6 + 8.4 + 4.2) (amount in kg) were the treatments with three replications. The 80:20 sewage sludge-to-press mud ratio was selected based on their complementary physicochemical properties. Sewage sludge provides high nutrient content but has a relatively dense structure and elevated heavy metal concentrations, whereas press mud contributes additional organic carbon and improves aeration and porosity. Preliminary characterization showed that this ratio produced an initial C/N ratio, which falls within the optimal range for precomposting and subsequent vermicomposting. This balance was essential to support microbial mineralization during preincubation and to create favorable growth conditions for *Eisenia fetida*. Samples were collected from each preincubation reactor at the beginning and end of the experiment.

#### 2.4.2. Vermicomposting Experiment

The obtained composts from initial mixtures of SS, PM, and SBB were shifted to vermicomposting reactors. The detailed specification of each vermicomposting reactor is already given in our previously published work [3]. Before the start of the vermicomposting experiment, 500 g of composting mixture was shifted to all vermireactors, and 20 mature earthworms having a total earthworm biomass of about 8.5 g were shifted to each vermireactor. The vermicomposting experiment was conducted in triplicate for each treatment at room temperature in dark conditions. During the 30 days of the vermicomposting process, different growth parameters of earthworms were recorded, and the vermicompost obtained was subjected to different analyses at the end of vermicomposting.

### 2.5. Activity of Earthworms and Chemical Analysis

#### 2.5.1. Growth of Earthworms

The number of cocoons, total earthworm biomass (g), change in weight (%), growth rate (mg/day/worm), reproduction rate (cocoons/day/worm), and survival rate (%) were among the growth parameters that were examined during the vermicomposting phase in each vermicomposting reactor. After every five days during the vermicomposting process, earthworms were removed from each vermireactor, manually sorted, numbered, cleaned with distilled water, and weighed [3].

#### 2.5.2. Chemical Analysis

All of the samples collected during preincubation and vermicomposting were subjected to chemical analysis using standard methods [12], such as elemental carbon and nitrogen analyses using a CN elemental analyzer (Flash EA 1112, Thermo Finnigan, Milan, Italy), organic matter content (loss on ignition in a muffle furnace at 550 °C for 6 h), and pH in H_2_O extract 1:2.5 *w*/*w* using a pH meter (PB-10, Sartorius AG, Göttingen, Germany). Following acid digestion, total heavy metal concentrations were measured on a dry-weight basis by Inductively coupled plasma mass spectrometry (ICP-MS; Agilent 7500a, Agilent Technologies, Santa Clara, CA, USA). A Fiber analyzer (F800, Hanon Instruments, Jinan, China) was used to determine the biological content (in terms of cellulose, hemicellulose, and lignin) [17].

#### 2.5.3. Bioaccumulation Factor (BAF)

The bioaccumulation factor was determined at the conclusion of the vermicomposting experiment using the standard methodology used in our earlier research [3]. Bioaccumulation factors (BAFs) were calculated for various trace elements, i.e., Cd, Cr, Cu, Mn, Pb, and Zn, by using the following equation (Equation (1)):

BAF_Me_ = (CMe earthworm)/(CMe vermicompost) (1)

where CMe earthworm is the total concentration of a selected metal in the earthworm body (mg.g^−1^) and CMe vermicompost is the total concentration of this metal in vermicompost (mg.g^−1^).

### 2.6. Statistical Analysis

The present study’s findings are the means of three replicates (*n* = 3). In order to assess the significant changes between treatments at the 0.05 percent significance level, this study used one-way analysis of variance (ANOVA). The same types of microcosms were categorized using Fisher’s Least Significant Difference (LSD) test based on the growth parameters of earthworms, nutritional elements, and heavy metals. All statistical analyses were conducted using OriginPro 8.0 and Statistica 8.1.

## 3. Results and Discussion

### 3.1. Morphological Assessment of SBB, Initial Mixture, and Final Vermicompost

**Figure 1:**
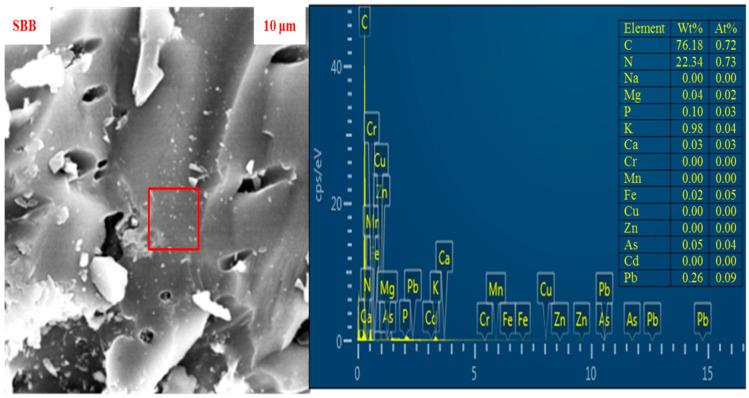
Scanning electron microscopy (SEM) images of sugarcane bagasse biochar (SBB) and corresponding energy-dispersive X-ray spectra (EDS) collected from the SEM region of SBB. □ sign shows the place where EDS was taken from.

**Figure 2:**
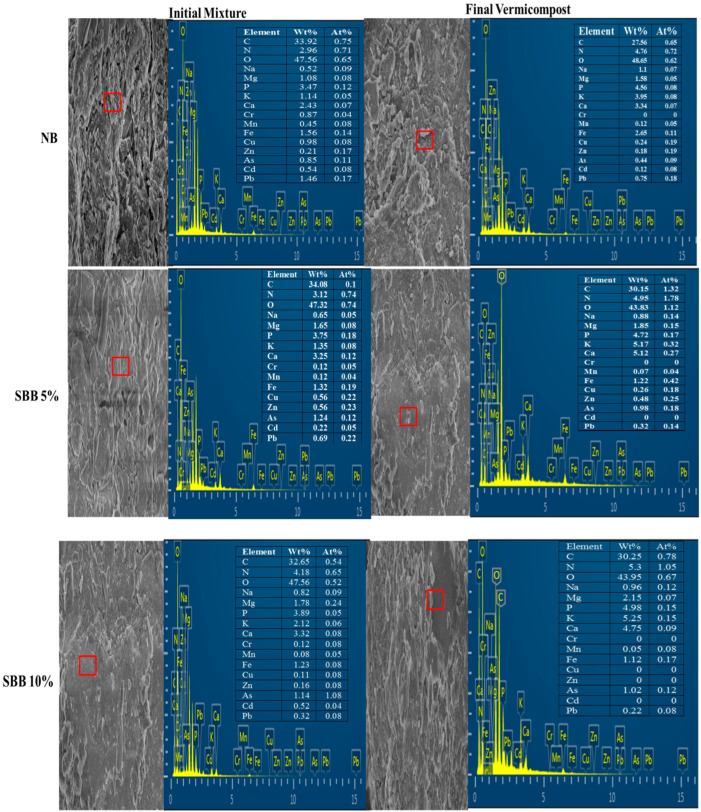
Scanning electron microscopy (SEM) images and corresponding energy-dispersive X-ray spectra (EDS) collected from the SEM region of the initial mixture and final vermicompost. □ sign shows the place where EDS was taken from.

Scanning electron microscopy (SEM) serves as an excellent instrument for examining the morphology of various solid particles. The SEM analysis revealed that SBB exhibited an irregular and fine pore structure (Figure 1). Additionally, the elemental composition of SBB was investigated using the SEM/EDS technique. The advantage of the SEM/EDS method lies in its non-destructive nature and its ability to provide quantitative data. The proximate and elemental analysis through EDS indicated that carbon and nitrogen were predominant on the surface of SBB, while other elements such as Mg, P, K, and Ca were present in variable amounts. The SEM/EDS findings for the initial mixture and the final vermicompost showed differences (Figure 2). The results indicated an increase in the concentration of macronutrients (N, P, K, Na, Ca, and Mg) in the final vermicompost, likely due to the mineralization process. Conversely, the concentration of various heavy metals decreased in the final vermicompost compared to the initial mixtures, which could be attributed to the reduced bioavailability of heavy metals through the application of SBB and possibly due to the bioaccumulation of heavy metals by earthworms during the vermicomposting of SS and PM. Moreover, porous structure and increased surface area of SBB, as observed in SEM images, likely enhanced microbial colonization, thereby facilitating nitrogen mineralization and phosphorus enrichment during preincubation and vermicomposting. This mechanistic linkage between morphological characteristics, improved nutrient availability, and enhanced *Eisenia fetida* performance during vermicomposting.

**Figure 3:**
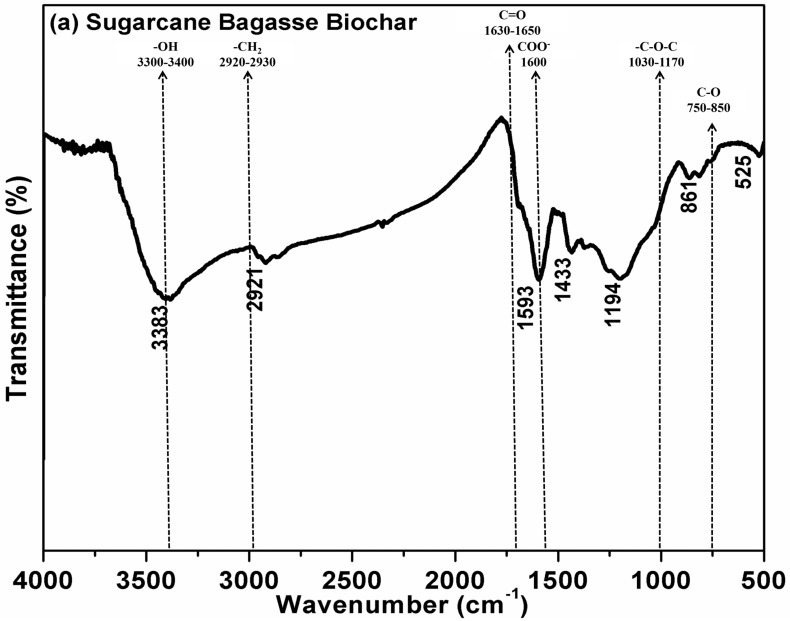
FT-IR spectra of sugarcane bagasse biochar (SBB).

Moreover, FTIR spectroscopy was employed to determine the chemical functional groups, as depicted in Figure 3. This technique is recognized as an effective method for identifying the chemical groups present in various materials [3]. The FT-IR spectra of the SBB displayed variations in absorbance intensity, which highlighted the presence of different chemical functional groups on the SBB surface. Drawing from previous studies and FT-IR analyses, the bands in the range of 3300–3400 cm^−1^ are indicative of (–OH) vibrations associated with hydroxyl groups in alcohol, phenol, and carboxyl functions. In contrast, the bands at 2920–2930 cm^−1^ are linked to the symmetric stretching of (–CH_2_) and (–CH_3_) in aliphatic compounds. The bands at 1600 cm^−1^ are related to aromatic C=O ring stretching, likely from –COOH. The bands at 1539 cm^−1^ may result from stretching vibrations of (C–O) in phenol. Additionally, the bands around 1430 cm^−1^ are attributed to the stretching of aromatic CO rings, reflecting their aromatic nature. The band near 1384 cm^−1^ could be due to soluble humus substances and represents (–COO–) stretching vibration, (–C–O–H–) bending vibration, or the absorption of aromatic compounds. Furthermore, there are peaks between 1000 and 1100 cm^−1^ in SBB, which correspond to alcohol C–O, aliphatic ether, or aromatic stretching. The bands at 1030–1170 cm^−1^ are associated with the stretching vibrations of (–C–O–C) in alcohols, ethers, and esters. Lastly, the bands at 750–850 cm^−1^ align with substrates containing (C–O) functional groups in carbonate compounds.

### 3.2. Effects of SBB on Earthworm Growth, Reproduction, and Survival

**Figure 4:**
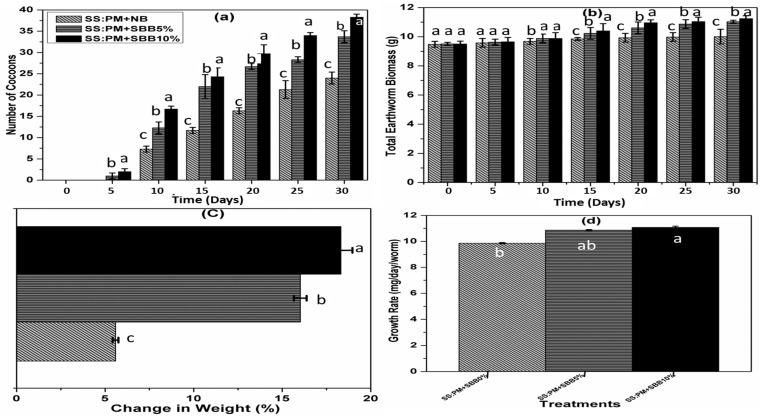

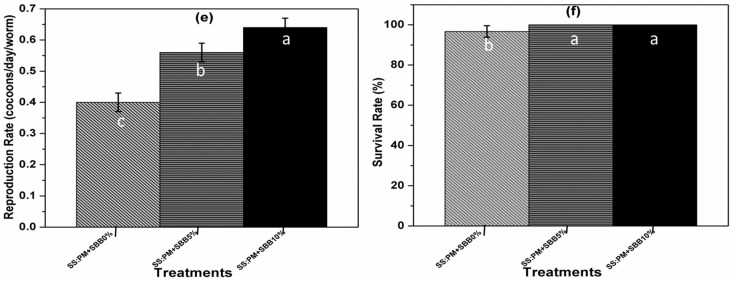
Earthworm growth, reproduction, and survival during vermicomposting: (**a**) number of cocoons, (**b**) total earthworm biomass, (**c**) change in weight, (**d**) growth rate, (**e**) reproduction rate, and (**f**) survival rate under different treatments. Different lowercase letters indicate significant differences among treatments at *p* < 0.05, based on one-way ANOVA followed by Fisher’s LSD test.

Earthworms are hermaphrodites, and when they become mature then form a ring-shaped clitellum that produces dense mucus to make a cocoon [18]. According to the results, the first cocoon appeared in SS + PM + 10% SBB on the 10th day of vermicomposting. At the end of vermicomposting, the average number of cocoons was 24 ± 1.4, 33.7 ± 1.4, and 38.3 ± 0.7 for the SS + PM + NB, SS + PM + 5% SBB, and SS + PM + 10% SBB, respectively (Figure 4). From the results, it is clear that the amendment of 5%SBB and 10% SBB before preincubation resulted in the improvement of cocoon number by 40% and 59%, respectively, in comparison to the mixture with no biochar. Similar results were obtained by Malińska et al. (2016), who observed 66% increase in cocoon production by the amendment of biochar as compared to the control [19]. The results showed that total earthworm biomass steadily increased in all treatments. At the end of vermicomposting, total earthworm biomass was 10 ± 0.1, 11 ± 0.3, and 11.2 ± 0.2 for the SS + PM + NB, SS + PM + 5% SBB, and SS + PM + 10% SBB, respectively. There was a positive change in the weight of earthworms in all the vermicomposting mixtures. At the end of vermicomposting, the change in weight was 5.5 ± 0.1, 16 ± 0.3, and 18.3 ± 0.6 for the SS + PM + NB, SS + PM + 5% SBB, and SS + PM + 10% SBB, respectively. This means that, in the mixtures amended with 5% and 10% SBB, the total weight of earthworms was better compared to the control. Reproduction and growth of earthworms are highly dependent on the quality and nature of organic material in the vermicomposting mixtures. At the end of vermicomposting, the growth rate was 9.8 ± 0.05, 10.8 ± 0.04, and 11 ± 0.09 mg/day/worm for the SS + PM + NB, SS + PM + 5% SBB, and SS + PM + 10% SBB, respectively. The growth rate and reproduction rate of earthworms were improved in the organic wastes, which have more available forms of nutrients. At the end of vermicomposting, the reproduction rate was 0.4 ± 0.03, 0.5 ± 0.05, and 0.6 ± 0.02 for the SS + PM + NB, SS + PM + 5% SBB, and SS + PM + 10% SBB, respectively. These results pointed out that the amendment of SBB to a mixture of SS and PM increased the *E. fetida* growth rate and reproduction rate. It is assumed that during vermicomposting, the biochar-compost matrix can attract trace elements, making them less bioavailable to earthworms, thus improving reproduction rate and growth rate. At the end of vermicomposting, the survival rate was 96.6%, 100%, and 100% for the SS + PM + NB, SS + PM + 5% SBB, and SS + PM + 10% SBB, respectively. A number of reasons, including enhanced overall porosity, higher water-holding capacity, increased nutrient retention capacity, availability of macro- and microelements, and improved microbial activity, may be responsible for this boost in earthworm development [12]. Although reduced metal bioavailability may have contributed to improved earthworm performance, biochar addition simultaneously modifies several key substrate properties, including nutrient availability, microbial activity, aeration, and moisture retention. These interacting factors were not independently controlled; therefore, the observed improvements in growth and reproduction cannot be attributed solely to metal stabilization. Instead, the positive response of *Eisenia fetida* likely reflects the combined effects of physicochemical enhancement and reduced toxicity.

Although this study demonstrates positive effects of sugarcane bagasse biochar on vermicomposting performance, only one particle-size fraction (<0.5 mm) was used. Biochar particle size is known to influence earthworm survival, substrate aeration, microbial activity, and organic matter degradation, yet this parameter remains largely overlooked in vermicomposting research. Therefore, the results presented here should be interpreted within the context of the specific particle size used, and future studies should systematically evaluate multiple particle size classes to determine their independent and interactive effects.

### 3.3. Effects of SBB on Heavy Metal Contents

**Figure 5:**
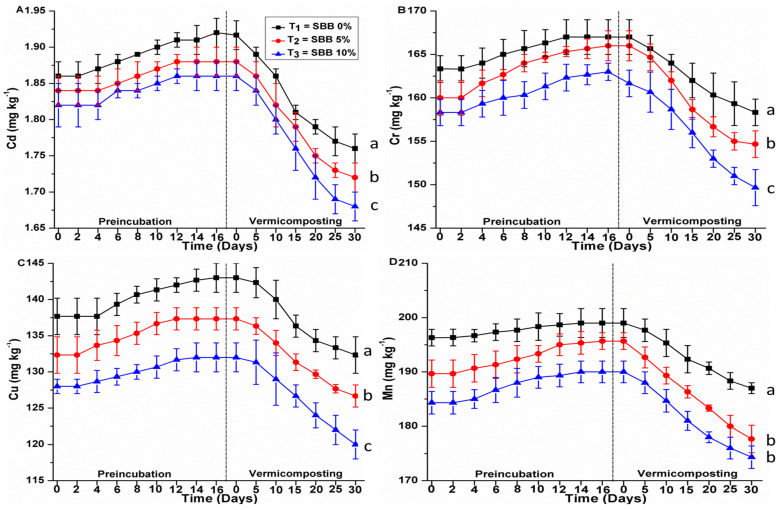

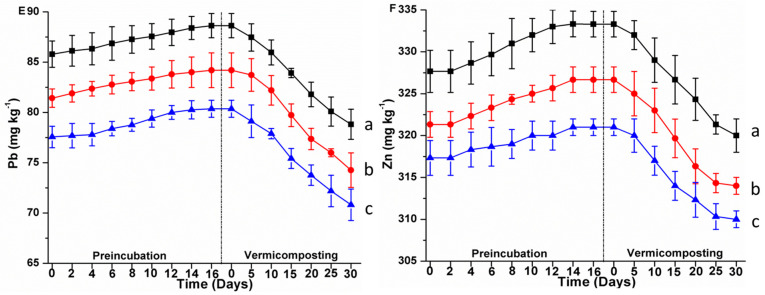
Dynamics of heavy metals during preincubation and vermicomposting phases. Different lowercase letters indicate statistically significant differences among treatments (*p* < 0.05), as determined by one-way ANOVA followed by Fisher’s LSD test at the end of the 30-day vermicomposting period.

Heavy metals in higher concentrations have detrimental effects on all types of living creatures. The toxicity level of heavy metals is dependent on various factors such as metal content, bioavailability, movement, and the heavy metal uptake process [20]. During vermicomposting of organic waste, earthworms consume organic resources, resulting in exposure of heavy metals to earthworms, and ultimately, trace elements are bioaccumulated in their bodies [21]. Results showed that adding 5% and 10% SBB reduced the amount of heavy metals in the finished vermicompost. This could be because the addition of SBB diluted the SS and PM combination. Additionally, a rise in earthworm growth and reproduction rates might be the cause of this. Figure 5 shows the heavy metal concentrations in the vermicomposts and preincubation combinations. The level of some heavy metals (Cd, Cr, Cu, Mn, Pb, and Zn) rose during preincubation, according to our findings. This might be because of organic matter weight loss, carbon dioxide release, and mineralization processes [12]. However, a decrease in heavy metals was observed when earthworms were put into each vermicomposting reactor. After the vermicomposting process, the overall mean (±SE) for Cd content was 1.7 ± 0.02 mg kg^−1^, 1.7 ± 0.02 mg kg^−1^, and 1.6 ± 0.03 mg kg^−1^ for SS + PM + NB, SS + PM + 5% SBB, and SS + PM + 10% SBB, respectively. LSD analysis exhibited that all treatment mixtures were significantly different (*p* < 0.05) from each other. Similarly, the overall mean (±SE) for Cr content was 158 ± 1.5 mg kg^−1^, 154 ± 1.5 mg kg^−1^ and 149 ± 2.0 mg kg^−1^ for SS + PM + NB, SS + PM + 5% SBB and SS + PM + 10% SBB, respectively. LSD analysis showed that at the end of vermicomposting, all treatment mixtures were significantly different (*p* < 0.05) from each other. Based on results the overall mean (±SE) for Cu content was 132 ± 2.5 mg kg^−1^, 126 ± 1.5 mg kg^−1^ and 120 ± 2.0 mg kg^−1^ for SS + PM + NB, SS + PM + 5% SBB and SS + PM + 10% SBB, respectively. LSD analysis showed that at the end of vermicomposting, all treatment mixtures were significantly different (*p* < 0.05) from each other. Our results showed that the overall mean (±SE) for Mn content was 187 ± 1.0 mg kg^−1^, 177 ± 2.5 mg kg^−1^, and 174 ± 2.0 mg kg^−1^ for SS + PM + NB, SS + PM + 5% SBB, and SS + PM + 10% SBB, respectively. LSD analysis showed that at the end of vermicomposting, all treatment mixtures were significantly different (*p* < 0.05) from each other. Furthermore, our results represented that the overall mean (±SE) for Pb content was 78.8 ± 1.5 mg kg^−1^, 74.2 ± 1.7 mg kg^−1^, and 70.8 mg kg^−1^ for SS + PM + NB, SS + PM + 5% SBB, and SS + PM + 10% SBB, respectively. LSD analysis showed that at the end of vermicomposting, all treatment mixtures were significantly different (*p* < 0.05) from each other. Similarly, the overall mean (±SE) for Zn content was 320 ± 2.0 mg kg^−1^, 314 ± 1.0 mg kg^−1^ and 310 ± 1.0 mg kg^−1^ for SS + PM + NB, SS + PM + 5% SBB and SS + PM + 10% SBB, respectively. LSD analysis showed that at the end of vermicomposting, all treatment mixtures were significantly different (*p* < 0.05) from each other. Moreover, a significant reduction in total content of Cd (5.3–7.6%), Cr (3.0–4.8%), Cu (3.8–6.2%), Mn (4.7–5.4%), Pb (8.1–8.6%), and Zn (2.3–2.3%) was observed during vermicomposting. When sewage sludge and sugarcane industrial wastes were vermicomposted together, Suthar (2010) reported a decrease in the content of heavy metals such as Cu, Fe, Zn, and Pb in the final vermicompost [22]. Earthworms and related microbial communities decreased metal movement throughout the vermicomposting process by enhancing enzymatic activity in both gut-associated processes (GAPs) and cast-associated processes (CASs) [3]. Although small reductions in total metal concentrations (3–8%) were observed, these values may partially reflect dilution effects from biochar addition and mass loss during decomposition. Therefore, detoxification should not be inferred from total concentration alone. Instead, the observed decreases in bioavailability factors provide stronger evidence of metal stabilization, as they indicate reduced uptake by *Eisenia fetida* and lower ecological risk. The current study’s findings showed that the vermicompost produced was safe for plant development since all of the heavy metals were below the maximum permissible metal concentrations set by various countries [3]. Although decreases in total metal concentrations were observed, these values alone cannot distinguish between true immobilization, dilution by biochar, or concentration changes resulting from organic matter loss. Because mass balance calculations were not performed, the observed reductions should be interpreted as concentration-based trends rather than evidence of mechanistic stabilization. Future studies should incorporate full mass balance assessments, including metal quantification in earthworm tissues, leachate, and residual solids, to accurately determine metal fate.

### 3.4. Bioaccumulation Factors (BAFs)

**Figure 6:**
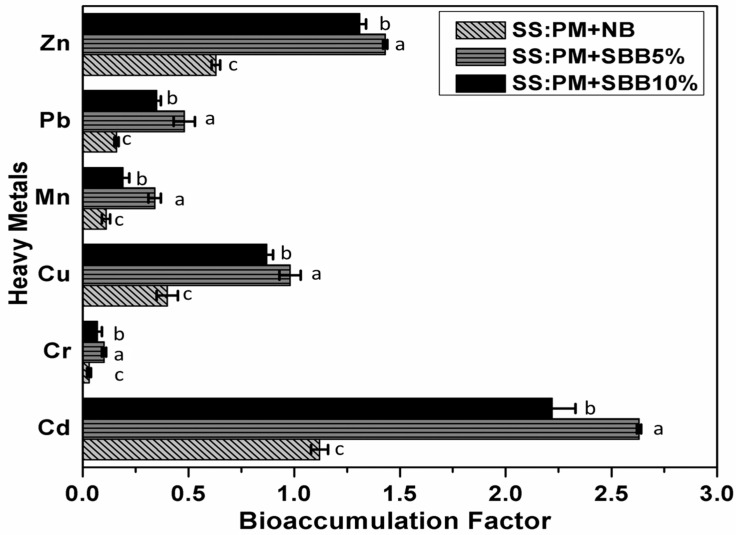
Bioaccumulation factor of heavy metals during vermicomposting. Different lowercase letters indicate significant differences among treatments at *p* < 0.05, based on one-way ANOVA followed by Fisher’s LSD test.

Earthworms may bioaccumulate various heavy metals from various environmental media, including polluted soils and heavy metal-contaminated waste, in their body tissues [12]. The mobile fractions of trace elements are either bioaccumulated in cutaneous tissues or bound to low molecular weight, cysteine-rich metal binding proteins like metallothionein (MTs), which maintain a strong attraction for trace elements like Hg, Zn, Ni, Co, Cu, and Cd, when earthworms consume organic wastes [23]. Moreover, Hsu et al. (2006) states that two methods are used to assess the trace elements that earthworms accumulate: the bioaccumulation factors (BAFs) and the bioconcentration factors (BCFs) [24]. Figure 6 shows the bioaccumulation factor of six heavy metals. At the end of vermicomposting, the total amount of trace elements (Cd, Cr, Cu, Mn, Pb, and Zn) in the earthworm body tissues were measured. According to our results for Cd, the BAFs values were 1.1 ± 0.04 (SS + PM + NB), 2.6 ± 0.01 (SS + PM + 5% SBB), and 2.2 ± 0.1 (SS + PM + 10% SBB). For Cr the BAFs values were 0.03 ± 0.01 (SS + PM + NB), 0.1 ± 0.01 (SS + PM + 5% SBB) and 0.07 ± 0.02 (SS + PM + 10% SBB). For Cu the BAFs values were 0.4 ± 0.05 (SS + PM + NB), 0.9 ± 0.05 (SS + PM + 5% SBB) and 0.8 ± 0.03 (SS + PM + 10% SBB). For Mn the BAFs values were 0.1 ± 0.02 (SS + PM + NB), 0.3 ± 0.03 (SS + PM + 5% SBB) and 0.1 ± 0.03 (SS + PM + 10% SBB). For Pb the BAFs values were 0.1 ± 0.01 (SS + PM + NB), 0.4 ± 0.05 (SS + PM + 5% SBB) and 0.3 ± 0.02 (SS + PM + 10% SBB). For Zn the BAFs values were 0.6 ± 0.02 (SS + PM + NB), 1.4 ± 0.01 (SS + PM + 5% SBB) and 1.3 ± 0.03 (SS + PM + 10% SBB). Khan et al. (2019), who noted bioaccumulation of Cd, Cr, Cu, Mn, Pb, and Zn during vermicomposting of sewage sludge by *E. fetida*, corroborate our findings [3]. Although reductions in bioaccumulation factors (BAFs) suggest decreased metal mobility in the vermicompost, bioaccumulation in earthworm tissues should not be interpreted as detoxification. Instead, it represents a redistribution of metals into biological compartments. This transfer may create potential trophic-transfer pathways for predators of *Eisenia fetida*; therefore, the ecological risk is shifted rather than eliminated. Future studies should evaluate metal burdens in earthworm tissues and assess potential risks to higher trophic levels.

### 3.5. Effects of SBB on Biological Contents

The samples of initial mixtures, precomposts, and vermicomposts were analyzed for lignocellulosic material. At the end of vermicomposting, cellulose was measured as 46 ± 0.5%, 42 ± 1.0%, and 41 ± 0.5% in SS + PM + NB, SS + PM + 5% SBB, and SS + PM + 10% SBB, respectively (Table 2). Our results showed that cellulose reduction from the ‘control’ was 16%, whereas it was 22% from SS + PM + 5% SBB and 23% from SS + PM + 10% SBB. According to results, the overall mean (±SE) for hemicellulose content was 25 ± 1.0%, 23 ± 0.5%, and 22 ± 1% for SS + PM + NB, SS + PM +5% SBB, and SS + PM + 10% SBB, respectively. Hemicellulose reduction from the ‘control’ was 17.3%, whereas it was 21.4% from SS + PM + 5% SBB and 23.9% from SS + PM + 10% SBB. At the end of vermicomposting, lignin content was measured as 16 ± 0.5%, 13 ± 1%, and 11 ± 1% in SS + PM + NB, SS + PM + 5% SBB, and SS + PM + 10% SBB, respectively (Table 2). The results showed that lignin reduction from the ‘control’ was 16%, whereas it was 28% from SS + PM + 5% SBB and 39% from SS + PM + 10% SBB. Throughout the vermicomposting process, the decrease in lignocellulosic content was significant in biochar-amended mixtures as compared to the control. The reduction in the lignocellulosic content during this study could also be due to the synergistic interactions of microflora, earthworm, and biochar, which ensued rapid degradation of more easily utilized organic substances, i.e., soluble sugars, hemicellulose, and cellulose [25].

### 3.6. Effects of SBB on pH and Macronutrients of the Composition

**Figure 7:**
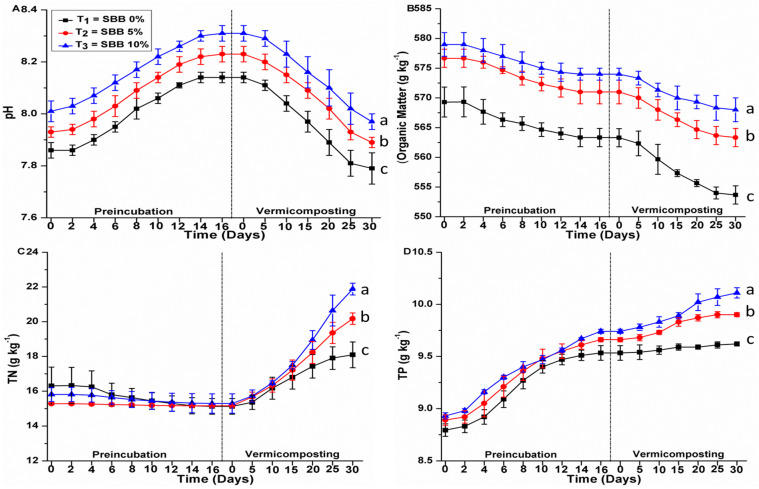

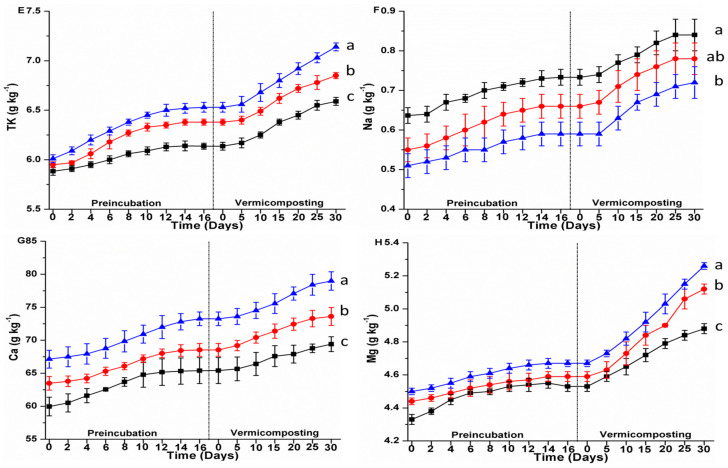
Dynamics of pH and macronutrients during preincubation and vermicomposting phases. Different lowercase letters indicate statistically significant differences among treatments (*p* < 0.05), as determined by one-way ANOVA followed by Fisher’s LSD test at the end of the 30-day vermicomposting period.

During the vermicomposting process, the pH of organic waste is a key parameter for efficient conversion of waste into vermicompost because earthworms can survive in a pH range of 5.0–9.0 [26]. At the end of the vermicomposting, pH values were 7.7 ± 0.06, 7.8 ± 0.02, and 7.9 ± 0.03 in SS + PM + NB, SS + PM + 5% SBB, and SS + PM + 10% SBB, respectively (Figure 7). The release of ammonia during the breakdown of organic matter may have caused the pH in all treatments to rise during preincubation, but the introduction of earthworms during vermicomposting tended to lower pH [3]. In a similar study, Fernández-Gómez et al. (2010) noted an increase in pH when fresh fruit and vegetable waste were vermicomposted [27]. However, the release of CO_2_ and the creation of organic acids by the combined activities of earthworms and microbes may be the cause of the pH drop during vermicomposting [3]. Additionally, during the vermicomposting of biowastes, earthworms can produce Ca and NH_4_-N through their intestine, which is crucial for maintaining the proper pH for neutralizing the phenolic and carboxylic groups of humic acids [28].

The results showed that organic matter (O.M) was reduced in all treatments during preincubation and vermicomposting. According to our results at the end of the vermicomposting, organic matter values were 553 ± 1.5, 563 ± 1.5, and 568 ± 2.0 in SS + PM + NB, SS + PM + 5% SBB, and SS + PM + 10% SBB, respectively (Figure 7). Khan et al. (2019) reported that the addition of different types of biochar during preincubation and vermicomposting can enhance the process of organic matter degradation [2]. Moreover, the addition of biochar during the composting of biowastes has positive impacts on the overall process by increasing the aeration, water holding capacity, and microbial biodegradation [29]. The current study’s findings indicate that organic matter degradation was higher in the mixes altered with SBB than in the control. Additionally, the microporous structure of SBB may promote the biodegradation of SS and PM mixes by giving microorganisms an appropriate habitat. Our findings are supported by those of Hait and Tare (2012), who noted that the mineralization and biodegradation of various organic wastes during the vermicomposting process may result in a significant decrease in organic matter, and that the rate of mineralization and biodegradation was higher in the presence of earthworms [30].

At the end of the vermicomposting, total nitrogen (TN) values were 18 ± 0.7, 20.1 ± 0.3, and 21.8 ± 0.3 in SS + PM + NB, SS + PM + 5% SBB, and SS + PM + 10% SBB, respectively (Figure 7). The average increase in total nitrogen in the final vermicomposts was by 10.9%, 32%, and 38.3% for the SS + PM + NB, SS + PM + 5% SBB, and SS + PM + 10% SBB, respectively, as compared to the initial mixtures. According to the results, TN for all mixtures increased during preincubation and vermicomposting. This increase in TN might be due to the mineralization of organic matter and the addition of earthworm nitrogenous excreta during the vermicomposting process [31].

According to the results, total phosphorus (TP) increased in all mixtures during vermicomposting. At the end of the vermicomposting process, TP values were 9.6 ± 0.02, 9.9 ± 0.02, and 10.1 ± 0.05 in SS + PM + NB, SS + PM + 5% SBB, and SS + PM + 10% SBB, respectively (Figure 7). The average increase in total phosphorus in the final vermicomposts was by 7.7%, 11.3%, and 15% for the SS + PM + NB, SS + PM + 5% SBB, and SS + PM + 10% SBB, respectively, as compared to the initial mixtures. In the mixtures amended with 5% and 10% SBB, the content of TP was significantly greater than that of SS + PM + NB (control). This increase in concentration of TP could be due to the presence of phosphorus in SBB. According to our findings, all treatments had higher phosphorus concentrations during preincubation. This might be because each treatment lost a net amount of organic carbon, hydrogen, oxygen, and dry biomass as CO_2_, H_2_S, and H_2_O [3]. Additionally, mineralization and the earthworms’ bacterial and fecal phosphatase activity may be responsible for the increase in phosphorus content during vermicomposting [3]. Moreover, Nayak et al. (2013) reported that a rise in TP during the vermicomposting of biowastes may result from the direct action of worm gut enzymes as well as indirect microbial activity [31].

At the end of vermicomposting, total potassium TK concentrations were 6.5 ± 0.03, 6.8 ± 0.03, and 7.1 ± 0.02 in SS + PM + NB, SS + PM + 5% SBB, and SS + PM + 10% SBB, respectively (Figure 7). The average increase in total potassium in the final vermicomposts was by 9.6%, 15.1%, and 21.4% for the SS + PM + NB, SS + PM + 5% SBB, and SS + PM + 10% SBB, respectively, as compared to the initial mixtures. Moreover, Bhat et al. (2015) also reported a significant increase in the concentration of TK during vermicomposting of different organic wastes [32]. This increase in concentration of TK during vermicomposting could be due to enhanced activity of microflora present in the earthworm gut [33].

At the end of the experiment, contents of Na were 0.8 ± 0.02, 0.7 ± 0.03, and 0.7 ± 0.04 in SS + PM + NB, SS + PM + 5% SBB, and SS + PM + 10% SBB, respectively (Figure 7). The average increase in Na content in the final vermicomposts was by 31.2%, 41.8%, and 41.1% for the SS + PM + NB, SS + PM + 5% SBB, and SS + PM + 10% SBB, respectively, as compared to the initial mixtures. Nayak et al. (2013) showed a similar rise in the concentration of Na during biowaste composting [31]. The results showed that the mixes altered with various ratios of SBB had lower concentrations of Na than the mixtures without biochar addition (control). This is a positive result since compost with a reduced Na^+^ ion content may help plants develop. On the other hand, compost with a greater concentration of Na^+^ ions may deteriorate soil structure and have detrimental effects on plant development. The findings demonstrated that during preincubation and vermicomposting, the concentration of Ca rose in all treatments over time. At the end of the experiment, contents of Ca were 69.4 ± 1.12, 73.6 ± 1.36, and 78.9 ± 1.4 in SS + PM + NB, SS + PM + 5% SBB, and SS + PM + 10% SBB, respectively (Figure 7). The average increase in Ca content in the final vermicomposts was by 3.3%, 15.9%, and 31.7% for the SS + PM + NB, SS + PM + 5% SBB, and SS + PM + 10% SBB, respectively, as compared to the initial mixtures. The present results in the current study are in accordance with previous study [12], which reported an increase in concentration of Ca during vermicomposting of different types of biowastes. According to our results, Mg concentration increased in all treatments with respect to time during preincubation and vermicomposting. At the end of the experiment, contents of Mg were 4.8 ± 0.03, 5.1 ± 0.05, and 5.2 ± 0.02 in SS + PM + NB, SS + PM + 5% SBB, and SS + PM + 10% SBB, respectively (Figure 7). The average increase in Mg content in the final vermicomposts was by 8.4%, 15.3%, and 20.9% for the SS + PM + NB, SS + PM + 5% SBB, and SS + PM + 10% SBB, respectively, as compared to the initial mixtures. Additionally, Nayak et al. (2013) noted that the mineralization of organic matter improved the concentration of magnesium in the composting mixes [31]. Other researchers reported similar findings (Singh et al., 2010), noted an increasing tendency in the final vermicompost’s magnesium content during the vermicomposting of various biowastes [34]. Although nutrient concentrations increased during vermicomposting, these changes largely reflect typical composting processes such as carbon mineralization and mass loss. Because the study did not include mechanistic measurements (e.g., microbial activity, nutrient sorption behavior, or CEC changes), biochar-specific effects cannot be isolated from standard composting dynamics. Therefore, the observed nutrient enrichment should be interpreted as the combined outcome of composting and biochar addition rather than a direct mechanistic effect of biochar alone.

### 3.7. Effects of SBB on Total Organic Carbon and Carbon to Nitrogen Ratio

At the end of the vermicomposting, total organic carbon (TOC) values were 326 ± 1.5, 331 ± 2.6, and 337 ± 2.0 in SS + PM + NB, SS + PM + 5% SBB, and SS + PM + 10% SBB, respectively (Table 2). According to the results, a reduction in TOC was observed during preincubation and vermicomposting. This decrease in TOC during the experiment indicates the rate of degradation of organic wastes. Furthermore, during vermicomposting, earthworms ingest different types of organic waste and decrease the concentration of organic carbon. This decrease in TOC during vermicomposting could be due to different factors such as microbial respiration and ingestion of available carbon as an energy source by the microbial communities and earthworms [15,32]. Similarly, Ravindran et al. (2008) also described the reduction in TOC from 55.1 to 21.1% during vermicomposting of animal fleshing solid waste [35].

At the end of the vermicomposting, C:N values were 18 ± 0.70, 16.4 ± 0.2, and 15.4 ± 0.2 in SS + PM + NB, SS + PM + 5% SBB, and SS + PM + 10% SBB, respectively (Table 2). A crucial metric that shows the extent of waste biodegradation and mineralization is the carbon-to-nitrogen ratio, or C:N ratio. The C:N ratio dropped in all treatment combinations during vermicomposting, according to the data. The reduction in TOC through microbial respiration in the form of CO_2_, the rise in TN in the form of mucus, and the stability of organic material by earthworm activity might all contribute to the decrease in the C:N ratio during vermicomposting [36].

## 4. Conclusions

This study demonstrates that sugarcane bagasse biochar (SBB) amendment significantly enhances both the vermicomposting process and final product quality. Among the tested amendment levels (0%, 5%, and 10%), the 10% SBB treatment produced the most favorable improvements in earthworm performance, nutrient enrichment, and heavy-metal stabilization. The results demonstrate the substantial improvement in *Eisenia fetida* performance, with 72.3% biomass increase and doubled cocoon production; accelerated lignocellulosic degradation, achieving 38.8% lignin decomposition; enhanced nutrient enrichment, with macronutrient concentrations increasing by 15–38%; effective heavy metal stabilization, reducing total metal content by 2.3–8.6% with decreased bioavailability; and improved maturity indices, including optimal C:N ratio (15.4) and reduced electrical conductivity. While these results demonstrate the strong potential of higher SBB inclusion, further studies using a broader range of concentrations are needed to identify the true optimal amendment rate. This integrated approach offers a viable solution addressing waste management challenges while contributing to circular economy principles through resource recovery and waste valorization. The findings provide a strong foundation for scaling up the biochar-enhanced vermicomposting system for industrial waste management and organic fertilizer production.

## Figures and Tables

**Table 1:** Characteristics of raw materials (all values are expressed on a dry-weight basis).

Parameters	Sewage Sludge (SS)	Press Mud (PM)	Sugarcane Bagasse Biochar (SBB)
pH	7.9 ± 0.02	7.3 ± 0.1	7.5 ± 0.1
O.M (g kg^−1^)	565 ± 3.0	272 ± 2.2	-
TOC (g kg^−1^)	336 ± 2.0	365 ± 4.0	317 ± 2.0
C/N (ratio)	22.8 ± 0.2	22.5 ± 0.2	21.9 ± 0.1
Macro elements (g kg^−1^)			
TP (g kg^−1^)	8.9 ± 0.07	0.72 ± 0.02	0.54 ± 0.01
TN (g kg^−1^)	14.6 ± 0.05	16.2 ± 0.05	14.5 ± 0.1
TK (g kg^−1^)	5.9 ± 0.04	3.8 ± 0.07	3.5 ± 0.03
Na (g kg^−1^)	0.6 ± 0.04	2.3 ± 0.02	2.1 ± 0.04
Ca (g kg^−1^)	65.8 ± 0.10	1.8 ± 0.03	1.4 ± 0.03
Mg (g kg^−1^)	4.4 ± 0.03	1.4 ± 0.01	1.1 ± 0.04
Trace elements (mg kg^−1^)			
Cd (mg kg^−1^)	1.8 ± 0.04	1.1 ± 0.02	0.5 ± 0.02
Cr (mg kg^−1^)	158 ± 2.0	98 ± 2.0	51 ± 1.5
Cu (mg kg^−1^)	132 ± 2.6	87.6 ± 0.1	45.3 ± 0.1
Mn (mg kg^−1^)	188 ± 2.5	256 ± 0.05	130 ± 0.1
Pb (mg kg^−1^)	82.5 ± 0.5	15.6 ± 0.1	8.8 ± 0.15
Zn (mg kg^−1^)	316 ± 3.6	289 ± 0.05	192 ± 0.1

Abbreviations: SS = sewage sludge; PM = press mud; SBB = sugarcane bagasse biochar; O.M = organic matter; TOC = total organic carbon; C/N = carbon-to-nitrogen ratio; TP = total phosphorus; TN = total nitrogen; TK = total potassium; Na = sodium; Ca = calcium; Mg = magnesium; Cd = cadmium; Cr = chromium; Cu = copper; Mn = manganese; Pb = lead; Zn = zinc.

**Table 2:** Characteristics of the initial mixtures, precomposts, and vermicomposts.

	SS + PM + 0% SBB	SS + PM + 5% SBB	SS + PM + 10% SBB
**Initial Mixture**			
Cellulose (%)	55 ± 1.0 ^a^	54 ± 0.5 ^a,b^	53 ± 0.5 ^b^
Hemicellulose (%)	29 ± 0.5 ^a^	28 ± 0.5 ^a,b^	27 ± 0.5 ^b^
Lignin (%)	19 ± 0.5 ^a^	18 ± 1.0 ^a,b^	17 ± 0.4 ^b^
TOC (g kg^−1^)	348 ± 3.0 ^b^	352 ± 1.5 ^a,b^	355 ± 1.5 ^a^
C/N (ratio)	21.4 ± 1.4 ^a^	23 ± 0.5 ^a^	22.4 ± 0.4 ^a^
**Precomposts**			
Cellulose (%)	51 ± 1.0 ^a^	49 ± 1.0 ^b^	47 ± 1.0 ^c^
Hemicellulose (%)	26 ± 0.5 ^a^	25 ± 1.0 ^a,b^	24 ± 0.5 ^b^
Lignin (%)	18 ± 1.0 ^a^	15 ± 0.5 ^b^	14 ± 1.0 ^b^
TOC (g kg^−1^)	343 ± 1.5 ^b^	348 ± 1.0 ^a^	350 ± 2.0 ^a^
C/N (Ratio)	22.7 ± 0.7 ^a^	22.9 ± 0.1 ^a^	22.9 ± 0.8 ^a^
**Vermicomposts**			
Cellulose (%)	46 ± 0.5 ^a^	42 ± 1.0 ^b^	41 ± 0.5 ^b^
Hemicellulose (%)	25 ± 1.0 ^a^	23 ± 0.5 ^a,b^	22 ± 1.0 ^b^
Lignin (%)	16 ± 0.5 ^a^	13 ± 1.0 ^b^	11 ± 1.0 ^c^
TOC (g kg^−1^)	326 ± 1.5 ^c^	331 ± 2.6 ^b^	337 ± 2.0 ^a^
C/N (Ratio)	18 ± 0.7 ^a^	16.4 ± 0.2 ^b^	15.4 ± 0.2 ^c^

Values are means ± SE (*n* = 3). Means in a column followed by different letters are different at *p* < 0.05, based on one-way ANOVA followed by Fisher’s LSD test.

## Data Availability

The data presented in this study are available on reasonable request from the corresponding author. The data is not publicly available due to ethical and privacy restrictions.
